# Ferrate (VI), Fenton Reaction and Its Modification: An Effective Method of Removing SARS-CoV-2 RNA from Hospital Wastewater

**DOI:** 10.3390/pathogens11040450

**Published:** 2022-04-09

**Authors:** Dušan Žabka, Barbora Konečná, Peter Celec, Monika Janíková, Nadja Ivašková, Ľubomíra Tóthová, Michal Tamáš, Andrea Butor Škulcová, Noemi Púček Belišová, Ivana Horáková, Paula Bímová, Ján Híveš, Jozef Ryba, Boris Klempa, Monika Sláviková, Juraj Kopáček, Ján Krahulec, Miroslav Gál, Tomáš Mackuľak

**Affiliations:** 1 Department of Environmental Engineering, Institute of Chemical and Environmental Engineering, Faculty of Chemical and Food Technology, Slovak University of Technology, Radlinského 9, 812 37 Bratislava, Slovakia; michal.tamas@stuba.sk (M.T.); xskulcova@stuba.sk (A.B.Š.); noemi.belisova@stuba.sk (N.P.B.); ivana.horakova@stuba.sk (I.H.); 2 Institute of Molecular Biomedicine, Faculty of Medicine, Comenius University, Sasinkova 4, 811 08 Bratislava, Slovakia; basa.konecna@gmail.com (B.K.); peter.celec@imbm.sk (P.C.); mon.janikova@gmail.com (M.J.); nadjaivaskova@gmail.com (N.I.); lubomira.tothova@imbm.sk (Ľ.T.); 3 Institute of Pathophysiology, Faculty of Medicine, Comenius University, Sasinkova 4, 811 08 Bratislava, Slovakia; 4 Department of Molecular Biology, Faculty of Natural Sciences, Comenius University, Ilkovičova 6, 842 15 Bratislava, Slovakia; jan.krahulec@uniba.sk; 5 Department of Inorganic Technology, Faculty of Chemical and Food Technology, Slovak University of Technology, Radlinského 9, 812 37 Bratislava, Slovakia; paula.bimova@stuba.sk (P.B.); jan.hives@stuba.sk (J.H.); 6 Department of Polymer Processing, Institute of Natural and Synthetic Polymers, Faculty of Chemical and Food Technology, Slovak University of Technology, Radlinského 9, 812 37 Bratislava, Slovakia; jozef.ryba@stuba.sk; 7 Institute of Virology, Biomedical Research Center of the Slovak Academy of Sciences, Dúbravská cesta 9, 845 05 Bratislava, Slovakia; boris.klempa@savba.sk (B.K.); virumona@savba.sk (M.S.); virukopa@savba.sk (J.K.)

**Keywords:** Fenton-like reaction, ferrate (VI), hospital wastewater, RNA, SARS-CoV-2, degradation

## Abstract

The outbreak of the coronavirus disease 2019 (COVID-19) raises questions about the effective inactivation of its causative agent, Severe Acute Respiratory Syndrome Coronavirus 2 (SARS-CoV-2) in medical wastewater by disinfectants. For this reason, our study of wastewater from a selected hospital evaluated several different advanced oxidation methods (Fenton reaction and Fenton-like reaction and ferrate (VI)) capable of effectively removing SARS-CoV-2 RNA. The obtained results of all investigated oxidation processes, such as ferrates, Fenton reaction and its modifications achieved above 90% efficiency in degradation of SARS-CoV-2 RNA in model water. The efficiency of degradation of real SARS-CoV-2 from hospital wastewater declines in following order ferrate (VI) > Fenton reaction > Fenton-like reaction. Similarly, the decrease of chemical oxygen demand compared to effluent was observed. Therefore, all of these methods can be used as a replacement of chlorination at the wastewater effluent, which appeared to be insufficient in SARS-CoV-2 removal (60%), whereas using of ferrates showed efficiency of up to 99%.

## 1. Introduction

More than 6500 different types of viruses are currently recognized [1] and about 200 species are capable to infect humans [2]. The World Health Organization (WHO) has reported that up to 12 million people die each year from infectious intestinal diseases and waterborne infections [3]. This problem is expected to be exacerbated by further population growth, climate changes, natural disasters, migration, globalization, and corresponding hygiene and waste management challenges [4].

Viruses or resistant types of microorganisms occurring in wastewater are in some cases responsible for serious diseases and it is important to pay more attention to their occurrence in the collection system (adenoviruses, noroviruses, rotaviruses or hepatitis A, influenza, Ebola virus etc.), not only during the current pandemic [4,5,6]. It is generally known that several commonly detected groups of viruses (rotaviruses, coronaviruses, adenoviruses, enteroviruses, noroviruses, polyomaviruses, etc.) may be also transmitted by wastewater [4,6,7].

The COVID-19 pandemic has currently affected more than 220 countries. At the 17th December 2021, there have been 273,270,000 confirmed cases of COVID-19, including 5,354,000 deaths reported to WHO. The virus is transmitted preferentially by droplet infection, can contaminate various surfaces where it can be infectious for some time and can also enter the sewage system by rinsing and washing textiles obtained from infectious individuals [8,9].

Thus, especially at a time of increased incidence of COVID-19 in the population, wastewater from health care facilities can represent a potential point source of this type of pollution, for example for municipal wastewater [10]. A study published by Yeo et al. confirms the possible transmission of SARS-CoV-2 by the fecal-oral route [11]. Up-to 50% of COVID-19 positive cases also suffer from diarrhea [8,12,13], with viral RNA detected in faeces [8,14]. In the context of new scientific reports on the spread of COVID-19 [15] and a possible further wave of infection, it is important to respond appropriately to prevent, for example, the transmission of potentially infectious SARS-CoV-2 virus into the sewer network by secretions from infectious patients (especially stool and urine) [8,11,12,13,15,16]. A study published by Medema et al. among other things points out the possibility of detecting the virus RNA directly in wastewater and a study by Pan et al. found viral RNA was present in faeces of 9 out of 17 investigated cases [17,18]. Moreover, the virus and its RNA can penetrate the sewer not only via stool and urine, but also by rinsing contaminated surfaces, for example during rain or when washing contaminated fabric and hands.

There is also strong evidence of multiplication of SARS-CoV-2 in the gut and infectious virus has occasionally been recovered from both urine and stool samples [19]. Woelfel et al. reported detection of SARS-CoV-2 in faeces of 8 out of 9 examined infected people [16]. Chen et al. detected the RNA in faeces of 12 out of 22 patients [20]. Lescure et al. described the presence of RNA in faeces in two out of five infected individuals [21]. Woelfel et al. and Wang et al. also tried to recultivate the virus from the stool [16,22]. Only Wang et al. was successful in isolating the infectious virus in cell culture in two out of four isolation attempts [22].

Another important factor is the different time of virus release from an infected individual by excrement and virus load [23,24]. Some viruses that are well spread by water are released from the human body in amounts of 10^2^–10^12^ copies/g of excrement) [4]. Pan et al. reported virus loads of 550 to 1.21 × 10^5^ copies per mL of stool [18]. Lescure et al. reported 10^6.2^–10^6.8^ and 10^7.4^–10^8.1^ genome copies per gram of stool in two patients [21]. Woelfel et al. observed the occurrence of viral RNA in 8 different patients and found that in one patient, the number of RNA copies during the peak of infection could reach 10^8^ per gram of stool [16]. The presence of the virus was subsequently confirmed in the urine of infected individuals [25]. However, one cannot exclude the possibility that wastewater from hospitals or healthcare facilities containing SARS-CoV-2 can be a point source of infection [10].

In terms of time, we do not know the period during which a given virus, influenced by various factors such as the presence of oxygen, particulate matter, chemical composition, microbial community, pH, temperature, etc., can remain infectious in wastewater [4,8]. Results obtained on the related SARS-CoV-1 virus suggest that these types of viruses may be infectious in wastewater for some period [26]. However, the virus is probably inactivated more rapidly than non-enveloped human viruses (e.g. adenoviruses, noroviruses, rotaviruses or hepatitis A) [4,6,8]. Heat, high or low pH, sunlight, and common disinfectants (such as chlorine-based) significantly accelerate its inactivation [3,4,6,8].

Wastewater from medical facilities is often treated by a combination of biological, physical, and chemical processes supplemented by a disinfection process [27,28]. These are mainly conventional biological treatment plants combined with disinfection based mainly on the application of chlorine gas or compounds based on it or germicidal UV emitters [27,28]. Due to several studies [10,29,30,31] dealing with the ability to treat wastewater from medical facilities, it is known that the effectiveness of biological (nitrification-denitrification), chemical (chlorination, coagulation) and mechanical treatment is limited and chemical and biological pollution can reach the aquatic ecosystem [10,28,32]. The ability to efficiently treat wastewater, in terms of chemical and biological pollution, is achieved by advanced oxidation processes (AOPs) and their various combinations or modifications [27,31,33,34,35,36].

The essential step is the production of free radicals, especially hydroxyl radical and various forms of reactive oxygen species (ROSs) [27,31,34,35,36]. In general, the most effective AOPs that are currently being extensively investigated for the removal of pathogenic microorganisms, viruses, and resistance genes include Fenton’s reaction (FR), Fenton-like reaction (FLR), Photo-Fenton’s reaction, photolysis and ozonation [31,34,35,36,37]. The Fenton reaction and its modifications have been described in several studies as a highly efficient process of inactivation of various types of viruses and antibiotic resistance genes capable of spreading through the aquatic environment [34,36,37,38]. For example, a study by Nieto-Juarez and Kohn (2013) investigated the possibility of using heterogeneous FR to inactivate MS2 coliphage virus. In addition to virus inactivation, the aim of the study was to observe possible adsorption on the surface of selected commercial iron oxides (α-Fe_2_O_3_, α-FeOOH, Fe_3_O_4_, Fe(OH)_3_) [39]. The virus under investigation adsorbed on all types of particles approximately equally. The authors of the study assume the influence of van der Waals forces or hydrophobic interactions.

In addition to these AOPs, the use of ferrate (VI) in wastewater purification and disinfection appears to be very promising [27,32,40]. Ferrate (VI) ions are very strong oxidizing agents that can remove wide range of micropollutants as well as pathogenic microorganisms, and viruses occurring in municipal wastewater from medical facilities [27,31,32,40].

Although RNA and intact, infectious virus are not the same thing and RNA concentrations probably cannot be used to simply predict risk, current data about degradation of viral RNA in wastewater by FR, FLR and ferrate (VI) are insufficient. In our work, the modified Fenton-like reaction with zerovalent iron (Fe^0^/H_2_O_2_/H_2_SO_4_ system) was tested. The effectiveness of this modification of the classical FR is in the intermediate generation of Fe^2+^ cations from Fe^0^ [27,32,41].

From the literature research, we are not aware of previous studies that concurrently monitor actual situation of SARS-CoV-2 presence in wastewater and subsequent experimental degradation of the virus using AOPs. Thus, our study is divided into two parts. The aim of the first part is to find out the occurrence of SARS-CoV-2 RNA in wastewater from a selected hospital during the culminating pandemic of COVID-19 in Slovakia. The aim of the second part of our research is to study possible degradation of real SARS-CoV-2 virus in model water and degradation of SARS-CoV-2 RNA in effluent wastewater taken from hospital wastewater treatment plant (HWWTP) by three different methods: Fenton reaction; modified Fenton reaction; and ferrate (VI). The obtained results indicate a high efficiency of all investigated degradation procedures to remove SARS-CoV-2 at the effluent from HWWTP.

## 2. Results

At first, the total amount of RNA in HWWTP was evaluated. The following figures summarize the results of quantification of total RNA before and after purification on HWWTP and show the ability of the oxidation procedures used to remove RNAs from wastewater from HWWTP.

**Figure 1:**
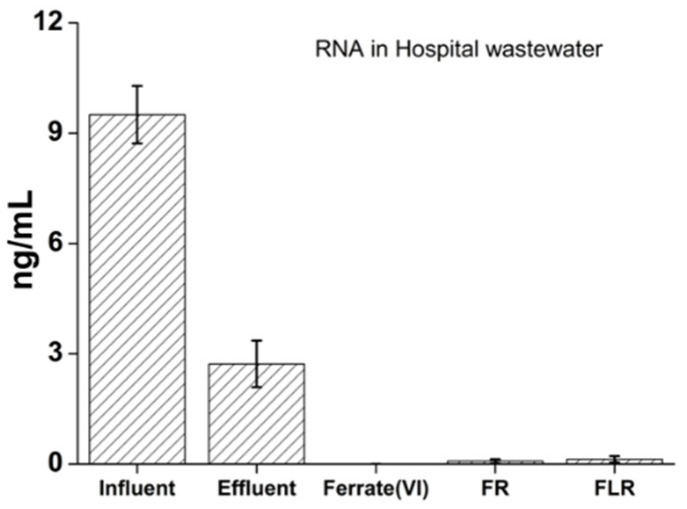
Comparison of the efficiency of total RNA removal using different oxidation processes at the effluent from hospital wastewater treatment plant as well as the efficiency of hospital wastewater treatment plant itself; FR—Fenton reaction, FLR—Fenton-like reaction.

In Figure 1, the amount of RNA found in influent and effluent of HWWTP together with amounts of respective nucleic acids after the post-treatment process by ferrate (VI), FR, and FLR is shown. For better visualization of efficiency of the individual AOPs, see Supplementary Figure S1, where the y-axis is plotted in logarithmic scale.

In Figure 1, it can be seen that the investigated HWWTP is able to remove total RNA with an efficiency of about 60% using a disinfection based on the application of chlorine gas at a concentration of about 6 mg/L of active chlorine. Similar findings, in terms of removal of RNA, are presented in another study [10], where the use of disinfection based on chlorinated agents is considered to be insufficient (800 g/m^3^ of sodium hypochlorite). However, all applied oxidation procedures were able to remove total RNA with efficiency more than 86% within 1 hour [10]. When comparing the efficiency of the investigated oxidation processes, it can be observed that the total RNA from the HWWTP effluent with the highest degree of efficiency was removed by the used ferrate (VI) and classical Fenton reaction (above 99%) (Figure 1). Although different reaction/degradation mechanism may apply depending on the structure of the target molecule, one of the most important reasons for the ferrate (VI) oxidation power is that the standard redox potential of ferrate (VI) is very high compared to other common disinfection agents. This potential increases with decreasing pH from +0.72 V in alkaline solutions to +2.2 V in acidic environment [42]. Ferrate (VI) is, therefore able to eliminate enveloped viruses such as SARS-CoV-2 by damaging the outer protein capsid and/or the interior nucleic acid [32,40]. However, to our best knowledge, the mechanism of RNA oxidation by ferrate (VI) has not been studied in detail. Oxidation of 2′-deoxyribonucleosides by potassium ferrate (VI) as a DNA chemical sequencing reagent and probe of its secondary structure was researched [43,44]. Degradation power of ferrate (VI) towards 2′-deoxyribonucleosides was decreasing in order deoxyguanosine > deoxycytidine = deoxythymidine > deoxyadenosine with guanosine and uridine bases as the main targets for oxidation of RNA by ferrate (VI), producing damages [44]. One electron or 2e^−^ transfer step with possible transfer of oxygen atoms to form Fe (IV) and corresponding nitro derivative is therefore probably initial step of oxidation mechanism of RNA using ferrate (VI). In addition, the purine ring is likely to open [43].

It is also necessary to emphasize the presence of active chlorine and chlorides in the wastewater at the effluent (Table 1) during degradation by individual oxidation processes. The possibilities of using iron and FR or FLR in the removal of micropollutants and pathogenic microorganisms, which have been investigated so far, have shown better results for FR and FLR [27,31]. However, the presence of free chlorine or chlorides can lead to a decrease in the effectiveness of FR and FLR. The generated hydroxyl radicals may partially react with the chlorine or chlorides present to form various Cl radicals (e.g., Cl radicals, which may also be involved in the degradation of RNA fragments present, but their oxidizing capacity is less than for the hydroxyl radical itself) [39,45]. It is known that hydroxyl radicals are not significantly scavenged by chloride ions unless the pH value is 4 or less [46]. Reactions between OH radicals and Cl_2_ or Cl ions can, therefore, have some negative effect on the overall efficiency of the Fenton reaction used and its modification under our conditions [9]. To conclude, all these results suggest that the investigated oxidation procedures have the ability to efficiently remove total RNA without dangerous chlorinated intermediates and thus be an effective ecological alternative.

**Figure 2:**
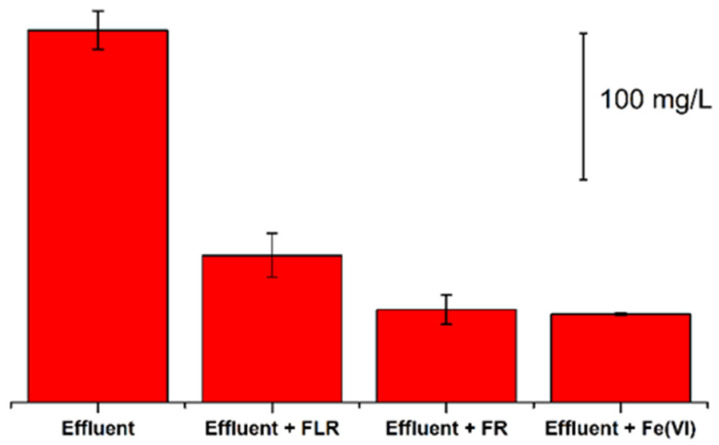
Chemical oxygen demand (COD) of the effluent of hospital wastewater before (1st column) and after the treatment with Fenton-like reaction (FLR), Fenton reaction (FR) and ferrate (VI) (Fe(VI)).

In Figure 2, the COD value found in effluent of HWWTP together with COD values after post treatment process by ferrate (VI), FR and FLR are shown. 

The oxidizing power of selected processes is directly proved by the decrease of COD value in examined effluent wastewater. The lowest efficiency of oxidation was observed for FLR, similarly as in case of the RNA removal (Figure 2). FR and ferrate (VI) showed approximately the same oxidizing power in COD removal. Limited oxidizing power of FLR may be caused, in particular, by the insufficient release rate of the Fe^2+^ ions from the iron shavings used in the reaction. These Fe^2+^ ions have the catalytic function and are necessary for the conversion of hydrogen peroxide to hydroxyl radicals (^●^OH). Another possible explanation of poorer degradation efficiency of FLR compared to other processes can be the oxidizing power of decomposing hydrogen peroxide. Modification of Fenton reaction may have certain limitations, mainly due to the gradual release of Fe^2+^ ions. Its efficiency could be increased, for example, by the gradual addition of hydrogen peroxide, which however, may not be simple from the technological point of view [27,31,37,38,41] 

**Figure 3:**
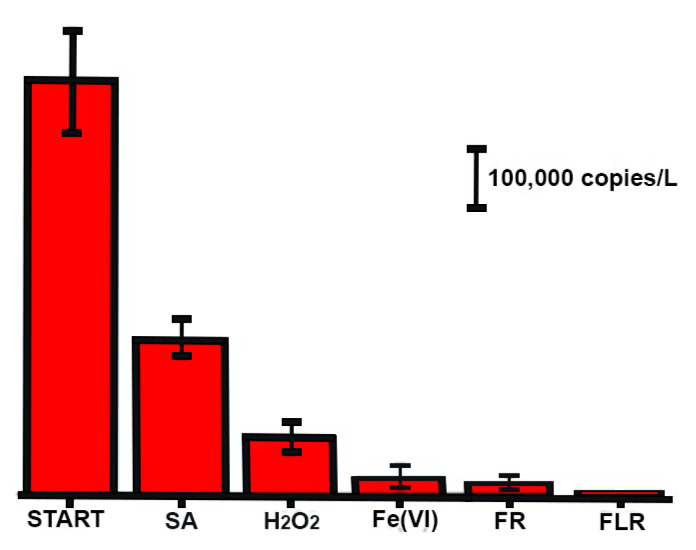
The amount of SARS-CoV-2 RNA in model water before (1st column) and after the treatment with sulfuric acid (SA), hydrogen peroxide (H_2_O_2_), ferrate (VI) (Fe (VI)), Fenton reaction (FR) and Fenton-like reaction (FLR).

In Figure 3, the amount of SARS-CoV-2 added into model water (labelled as START in Figure 3) together with amount of SARS-CoV-2 after all treatment processes are shown. To evaluate the effect of hydrogen peroxide and sulfuric acid used in FR and FLR, the oxidizing power of these pure chemicals was also separately tested. In case of sulfuric acid, the model water was acidified to pH 3 with 98% H_2_SO_4_ and in the case of hydrogen peroxide the same quantity was used as with FR and FLR. It can be seen from the Figure 3, that sulfuric acid and hydrogen peroxide effectively removed more than 50% of SARS-CoV-2 from model water. However, investigated oxidation processes showed much higher efficiency, above 90%. In model water high efficiency of oxidation by FLR can be seen, since hydrogen peroxide is not consumed for oxidation of other contaminants presence in real wastewater.

**Figure 4:**
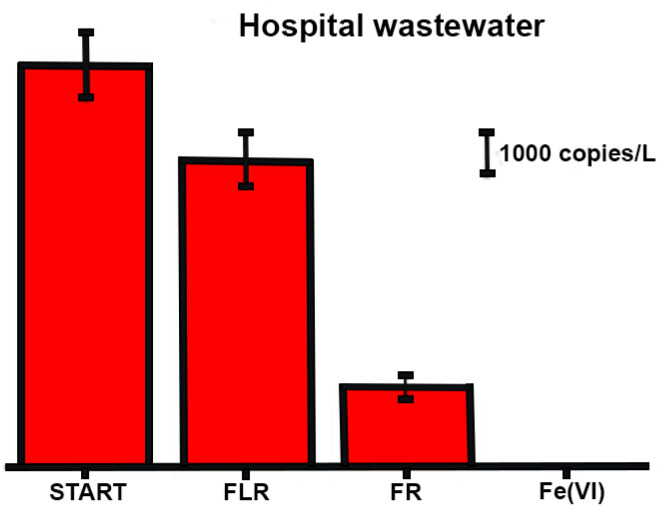
The amount of SARS-CoV-2 RNA copies in effluent from hospital wastewater (1st column) and after the treatment with Fenton-like reaction (FLR), Fenton reaction (FR) and ferrate (VI) (Fe (VI)).

The significantly better ability to remove SARS-CoV-2 RNA from model water compared to hospital wastewater can be attributed to the fact that hospital wastewater, unlike model water, contains a large number of other substances, such as proteins, fats, polysaccharides, drugs and their metabolites and the like [28], with which ^●^OH radicals and ferrate (VI) can react and thus relatively reduce their efficiency of the SARS-CoV-2 RNA removal process (Figure 3 and Figure 4).

The application of chlorine gas (6 mg/L) is only partially able to reduce SARS-CoV-2 RNA. On the other side, we were able to achieve the significantly higher degradation of SARS-CoV-2 RNA from HWWTP using mentioned AOPs methods and ferrate (VI). Figure 4 describes the ability of selected oxidation processes to remove SARS-CoV-2 RNA directly from the HWWTP effluent. The highest removal efficiency was achieved by ferrate (VI) and then by FR, which were able to reduce the amount of RNA detectable by RT-PCR by 99% and 80%, respectively. The lowest removal efficiency was observed for FLR. All these observations are in good correlations with results in model and influent wastewater.

Ferrate (VI) and the Fenton reaction are efficient oxidative processes [27,32] that are able to remove various viruses capable of spreading through wastewater. Therefore, both methods are expected to also remove SARS-CoV-2 and its RNA in case of occurrence. The modified Fenton reaction (Fenton-like reaction) applied in our study used waste iron shavings as an iron source [27,33]. FLR showed lower efficiency in removing total RNA from wastewater than FR, which has already been observed for the degradation of different groups of micropollutants in several studies [27,33]. Compared to the classical Fenton reaction, however, the modification produces OH radicals gradually, which is associated with the gradual release of Fe^2+^ ions into the reaction [27]. We have verified the potentially inhibitory effect of RNA eluate after using previously iron for degradation of SARS-CoV-2. We have run real time PCR for the detection of human mitochondrial DNA (Figure S1). Human DNA eluate was mixed in 1:1 ratio with either distilled water or with model water eluate, which was purified using iron. We have observed no CT value increase, which supports the statement, that Fe was completely filtered out, therefore it does not inhibit the reaction.

The advantage of FLR procedure for removing RNA compared to using ferrate (VI) and classical FR is that it is a little less expensive (Table 2) [27,31]. The economic costs of using FLR compared to FR are about 20–30% lower (Table 2). In laboratory conditions, this may not make a big difference, but in the case of deployment for a hospital treatment plant, the annual costs could already be an interesting item in the hospital’s budget. Estimating the flow of 90 m^3^ of wastewater per day, i.e., roughly 33,000 m^3^ per year this represents the difference—a rough estimate—16,000 €/year. The important parameter for the costs calculations is wastewater flow. If the flow is higher—in the case of large hospitals—the cost differences between FLR and FR are also higher.

In general, it can be said that the effort to modify the FR is mainly to reduce the cost of the process. In our study, we were focused on the inactivation of SARS-CoV-2 virus, but also directly on the removal of total RNA (viral RNA included). This is due to possible concerns about the presence of the virus as well as its potentially infectious RNA in wastewater from healthcare facilities even after disinfection [10]. The effect of the investigated FLR and FR in removing total viral RNA or viruses themselves in the effluent can be compared to the effects of strong disinfectants, both due to low pH (around 3) and the presence of generated hydroxyl radicals capable of destroying the virus envelope or nucleic acids themselves [34,36,37]. 

In Table 2, costs of individual technologies used for hospital wastewater post-treatment are estimated. However, it is clear that technology costs will decrease as the amount of post-treated wastewater increases. This study confirmed the significant ability of Fenton reaction and ferrate (VI) to sufficiently eliminate a wide range of RNA fragments. FLR shows less removal efficacy especially in the case of hospital wastewater (Figure 4) compared to these methods, however financial costs of this technology are ca 20% and even 40% lower compared to ferrate (VI) and FR, respectively (Table 2). Generally, the processes used are neither technically demanding nor very expensive. Despite WHO activities, education of people on the management and treatment of hospital waste is insufficient. Other existing methods for removing RNA, virus and "antibiotic-resistant bacteria" from wastewater, such as chlorine or ozone oxidation, are more expensive or produce hazardous intermediates, especially chlorination and ozonation [10,27,28,31,35].

The costs of treatment technologies data presented in the table were calculated on the basis of chemical prices obtained from their Slovak producers, priced for the annual operation of a treatment plant located in the surveyed hospital with a maximum wastewater flow—90 m^3^ per day. Given the reported environmental stability of coronavirus, the faeces- and sewage-derived transmission routes may be of importance to prevent unprecedented spread of COVID-19 particularly in developing countries. However, so far, limited number of studies detected infectious SARS-CoV-2 even in human faeces, whereas a number of virus RNA copies were identified in both faeces and sewage specimens [22,47]. Since, uncertainty remains in the possibility of this transmission pathway, further investigation is warranted in future studies, for example, by increasing the number of specimens, examining the effectiveness of methods for virus viability test, considering the patient medical history, and so forth [48,49].

## 3. Materials and Methods

### 3.1. Characterization of the Investigated Hospital, Its WWTP and Model Tap Water

In this study, wastewater from The Central Hospital, Slovakia (ca 500 permanent hospital beds, from which 200 was COVID19 infected patients) was investigated. This hospital is equipped with its own mechanical pretreatment of wastewater, classic nitrification and disinfection with gaseous chlorine with concentration of active chlorine of 6 mg/L. Before the infectious water enters the treatment plant building, the water flows through the control channels. It flows to the combs which are divided into right and left channels, while only one of them always works in real time. Here the water gets rid of coarse impurities and then goes through a channel to the tank where it gets rid of sand and gravel and then biologically purified with bacteria. Sludge settles to the bottom. Excess water, partly with the sludge, goes to the settling tank and here the excess sludge settles to the bottom again and the water passes to the mixing tank where it is chlorinated to prevent the survival of infectious substances. Subsequently, the water flows through the labyrinth, (6 hours) to prolong the chlorination effect and thus ensure the non-infectivity of the water. The hospital is then directly connected to the collection system. Basic characteristics of the effluent wastewater are listed in Table 1.

Tap water with pH = 7.33, conductivity = 58.4 mS/m, COD = 0.74 mg/L, chlorites less than 0.05 mg/L, chlorides = 30.52 mg/L, chlorates less than 0.05 mg/L, nitrates = 12.76 mg/L, sulphates = 41.42 mg/L served as a model water for the virus experiment.

### 3.2. Wastewater Sampling and Analysis

The wastewater samples (influent and effluent) were collected from the sampling point by an automatic sampling device from 7 to 9 a.m., decanted samples in 15-minute intervals with total volume of 1800 mL. The samples were collected into plastic bottles and were cooled (4 °C) immediately after the sampling. The influent water was collected before the inlet into WWTP and the effluent wastewater for degradation procedures was sampled before the inlet into the collection system. Sampling personnel wore standard personal protective equipment (PPE) for wastewater sampling, such as long pants, steel capped boots, hard hats, safety glasses and gloves to minimize potential exposure to infectious SARS-CoV-2. 

### 3.3. Chemicals and Instruments 

All the chemicals used in degradation of the effluent wastewater of the HWWTP by FR and FLR (iron sulfate (FeSO_4_·7H_2_O, 98%), hydrogen peroxide (H_2_O_2_) 30% solution, sulfuric acid (H_2_SO_4_) and sodium hydroxide (NaOH)), were obtained from Lachema Company (Brno, Czech Republic), and had p.a. purity. The aqueous solutions were prepared from demineralized water, pH was determined with a pH probe (Sentek, Braintree, United Kingdom).

Potassium ferrate (VI) was electrochemically prepared as described in our previous report [27]. Briefly: anode and counter electrode was composed of mild steel class 11. The cathodic and anodic compartments were divided by a diaphragm. The mass fraction of KOH was less than 70% (w/w). 

### 3.4. Degradation Methods Description

Generally, the optimal experimental conditions of analysis for all technologies in this study were used on the basis of our optimization experiments, our previous studies as well as one the previous studies published by other authors [27,31,50].

#### 3.4.1. Ferrate (VI)

Solid potassium ferrate (VI) of 33% purity was pressed to form a pellet weighting 125 mg [51]. Then 100 mL of untreated wastewater was added into a 100 mL conical tube and one pellet of ferrate (VI) was subsequently added and whole sample was vigorously mixed. The pH of the medium was equal to the pH of wastewater. After a 20 min of reaction, coagulate was filtered out and the concentrations of RNA fragments were determined in the filtrate. Since the study focused on the possibility of removing the virus RNA from the aqueous component and no coagulate is formed during FR and/or FLR, the coagulate formed in the degradation process by ferrate (VI) was removed by filtration before measuring RNA concentration. Experiments were repeated three times and the presented results are the mean values obtained from the measurements. 

#### 3.4.2. Classical FR Experiments (FeSO_4_·7H_2_O and H_2_O_2_)

We used the following procedure for the degradation experiment: the required amount of FeSO_4_·7H_2_O (27.343 mg, 98% purity) and H_2_O_2_ (0.08 mL, 30%, w/w) was added to a beaker (100 mL) containing 50 mL of wastewater at a pH of 3 (acidified with H_2_SO_4_, 98% w/w). The weight ratio of H_2_O_2_:FeSO_4_ was 875:500 mg/L. The homogeneous mixture was then stirred vigorously with an electromagnetic stirrer at 300 rpm. The reaction was continued for 60 min at 20 °C under daylight conditions. The reaction mixture was then neutralized with 20% of NaOH. Experiments were repeated three times and the presented results are the mean values obtained from the measurements.

#### 3.4.3. Fenton-Like Reaction Experiments (FLR-system Fe^0^/H_2_O_2_/H_2_SO_4_)

The procedure is described in detail elsewhere [27]. Briefly: iron shavings were prewashed and activated by immersion into a sulphuric acid solution to remove the surface layer of oxidized iron. Concentrated H_2_SO_4_ (5 μL), 0.3 g of activated iron shavings and H_2_O_2_ (0.16 mL, 30%, w/w) were then added to the flask with 50 mL of wastewater while the solution was constantly stirred with an electromagnetic stirrer. The pH of the medium was equal to 3. The mixture was then stirred for 60 min at 20 °C under daylight conditions. The reaction mixture was then neutralized with 20% of NaOH. Experiments were repeated three times and the presented results are the mean values obtained from the measurements.

### 3.5. Virus Propagation and Inactivation

We used SARS-CoV-2 strain Slovakia/SK-BMC5/2020 available at https://www.european-virus-archive.com/virus/sars-cov-2-strain-slovakiask-bmc52020 (accessed on 1 December 2021). The virus was propagated on VeroE6 cells and quantified by a plaque assay. For the experiments performed inside of biosafety level 3 laboratory, virus was diluted to contain 10,000 plaque forming units per mL of culture medium (PFU/mL) and inactivated at 60 °C for 30 min by using a water bath. After heat treatment, samples were frozen at −70 °C for later analyses.

### 3.6. Isolation and Quantification of Nucleic Acids

Hospital wastewater samples as well as model waters were processed according to [52]. Model waters were tap water samples spiked with a known concentration of SARS-CoV-2 virus strain Slovakia/SK-BMC5/2020 (https://www.european-virus-archive.com/virus/sars-cov-2-strain-slovakiask-bmc52020-fd accessed on 1 December 2021). The pellets were then applied for nucleic acids isolation using Trizol reagent. Qubit Fluorometer together with Qubit RNA HS Assay Kit (Thermo Fisher Scientific, Waltham, MA, USA) were used for the quantification of total RNA in the final samples according to standard Qubit protocol. The presence of SARS-CoV-2 RNA were assessed using QuantiTect SYBR Green RT-PCR kit (Qiagen, Hilden, Germany) with primers specific for N1 gene (F: GAC CCC AAA ATC AGC GAA AT; R: TCT GGT TAC TGC CAG TTG AAT) and N3 gene (F: GGG AGC CTT GAA TAC ACC AAA; R: TGT AGC ACG ATT GCA GCA TTG) as described previously [53]. Each 10 µL reaction mix contained QuantiTect SYBR Green PCR Master Mix (Qiagen, Hilden, Germany)—5 µL, 500 nM of forward and reverse PCR primers for N1 or N3 gene, QuantiTect RT Mix (Qiagen, Hilden, Germany)—0.1 µL, RNA template—1 µL and 2.9 µL nuclease free water. One-step real-time RT-PCR was performed on an Eppendorf realplex4 Mastercycler epgradient S (Eppendorf, Hamburg, Germany) with thermocycling conditions set at 50 °C for 30 min for reverse transcription, 95 °C for 15 min for initial denaturation, 45 cycles of amplification: 95 °C for 15 s, 55 °C for 30 s and 65 °C for 30 s, and followed by melting curve analysis. Presence of specific bands was checked using electrophoresis. All experiments were carried out in triplicate and the presented results are the mean values obtained from the measurements.

## 4. Conclusions

In our study, we focused on the ability of selected oxidation procedures to remove SARS-CoV-2 RNA from wastewater after treatment on HWWTP. There were 200 hospitalized infectious individuals with confirmed COVID-19 in the hospital during sampling. It was found that the investigated treatment plant (even when using 6 mg/L active chlorine) shows a total RNA removal efficiency of only 60%. First, a virus sample was tested in model water while being observed, that the highest removal efficiency was achieved using FLR, followed by FR and ferrate (VI).

The oxidation procedures investigated in our study showed a high efficiency of viral RNA removal (efficiency above 86%) in real wastewater treatment. In order to compare the efficiency of individual processes for the removal of viral RNA from HWWTP, it is possible to rank the procedures in terms of the highest efficiency as follows:

Ferrate (VI) > Fenton reaction > FLR

The identical efficiency was observed in SARS-CoV-2 RNA removal from HWW.

The effectiveness of the individual procedures in monitoring the values of COD can also be observed and it is possible to rank the oxidation procedures to the same order, so ferrate (VI) appear to be most effective. From the point of view of the efficiency of the tested oxidation processes, it can be stated that in real wastewater, where oxidation of several types of compounds occurs, ferrate (VI) appears to be the most effective compared with FLR, which is not sufficient to oxidize various contaminants that were not presented in the model water.

Long-used disinfection procedures, preferred by the WHO, may not strictly lead to the complete degradation of RNA from hospital wastewater. Although the fact, whether viral RNA is infectious at the specific point in wastewater or not is still unclear, removal of RNA ensures that any potentially harmful biological material is eliminated and leads to complete disinfection/inactivation of the viruses as proposed by WHO. Increasing number of chlorine-based preparations used can also lead to the formation of intermediates that are more toxic than the parent compounds. It is therefore necessary to consider the disinfection procedures and standards used, and to improve the treatment strategy for these types of wastewaters in the future.

## Figures and Tables

**Table 1:** Basic characteristics of the studied effluent wastewater.

Institution/Parameter	Central Hospital (Bratislava, Slovakia) - Effluent
Date of analysis	20 November 2020
Flow (m^3^/day)	90
Suspended solid	30g/person/day
Method of wastewater pretreatment	Nitrification/chloration (gas Cl_2_)
COD ^1^ (mg/L)	253
BOD ^2^ (mg/L)	66
pH	7.51
Cl_2_ (mg/L)	5.1
Cl^−^ (mg/L)	92
NH_4_-N (mg/L)	3.5
NO_3_-N (mg/L)	10
Temperature (°C)	11

^1^ Chemical oxygen demand; ^2^ Biochemical oxygen demand

**Table 2:** Estimated costs of individual technologies used for hospital wastewater post-treatment [31].

Technique	Price (EUR/m^3^)
HWWTP only nitrification	0.8–1.1
HWWTP nitrification and disinfection	0.9–1.3
Fenton reaction	1.2–1.5
Fenton-like reaction	0.7–1.0
Ferrate (VI) (33% purity)	1.0–1.1

## Data Availability

The data presented in this study are available within this article and Supplementary Material.

## Supplementary Materials

The following are available online at https://www.mdpi.com/article/10.3390/pathogens11040450/s1, Figure S1: Elimination of RNA fragments from hospital effluent wastewater by ferrate (VI), Fenton reaction, and Fenton-like reaction; y axis in logarithmic scale for better visibility.
