# Supplementary material for: Ferrate (VI), Fenton Reaction and Its Modification: An Effective Method of Removing SARS-CoV-2 RNA from Hospital Wastewater
Source: Pathogens. 2022 Apr 9;11(4):450. doi: 10.3390/pathogens11040450 (PMC9027194; doi:10.3390/pathogens11040450)
Supplement: Supplementary file 1 [file pathogens-11-00450-s001.zip › pathogens-1546133-supplementary.pdf]

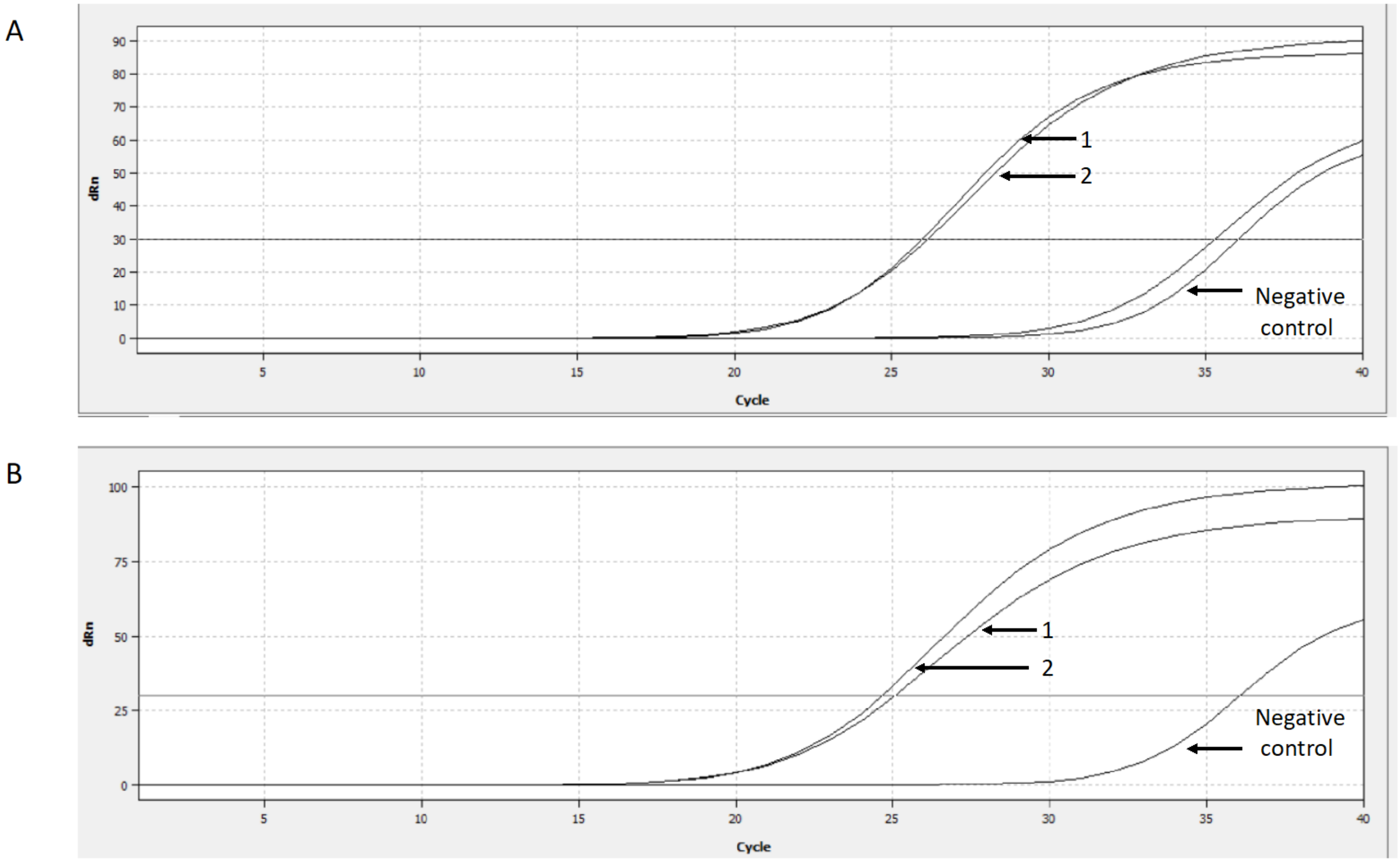

Figure S1: PCR targeting mitochondrial D-loop gene. Templates are 1:1 mixtures of human DNA eluate and (1) distilled water, (2) model water eluate, which contained SARS-CoV-2 and was purified using iron. A – example 1, B – example 2.
